# Pre-pregnancy weight, gestational weight gain, and the gut microbiota of mothers and their infants

**DOI:** 10.1186/s40168-017-0332-0

**Published:** 2017-09-04

**Authors:** Maggie A. Stanislawski, Dana Dabelea, Brandie D. Wagner, Marci K. Sontag, Catherine A. Lozupone, Merete Eggesbø

**Affiliations:** 1 Colorado School of Public Health, Colorado, Aurora USA; 2 University of Colorado School of Medicine, Colorado, Aurora USA; 3 Department of Environmental Exposure and Epidemiology, Norwegian Institute of Public Health, PO Box 4404, Oslo, Norway; 4 VA Eastern Colorado Health Care System, Denver, CO USA

## Abstract

**Background:**

Recent evidence supports that the maternal gut microbiota impacts the initial infant gut microbiota. Since the gut microbiota may play a causal role in the development of obesity, it is important to understand how pre-pregnancy weight and gestational weight gain (GWG) impact the gut microbiota of mothers at the time of delivery and their infants in early life. In this study, we performed 16S rRNA gene sequencing on gut microbiota samples from 169 women 4 days after delivery and from the 844 samples of their infants at six timepoints during the first 2 years of life. We categorized the women (1) according to pre-pregnancy body mass index into overweight/obese (OW/OB, BMI ≥ 25) or non-overweight/obese (BMI < 25) and (2) into excessive and non-excessive GWG in the subset of mothers of full-term singleton infants (*N* = 116). We compared alpha diversity and taxonomic composition of the maternal and infant samples by exposure groups. We also compared taxonomic similarity between maternal and infant gut microbiota.

**Results:**

Maternal OW/OB was associated with lower maternal alpha diversity. Maternal pre-pregnancy OW/OB and excessive GWG were associated with taxonomic differences in the maternal gut microbiota, including taxa from the highly heritable family Christensenellaceae, the genera *Lachnospira*, *Parabacteroides*, *Bifidobacterium*, and *Blautia*. These maternal characteristics were not associated with overall differences in the infant gut microbiota over the first 2 years of life. However, the presence of specific OTUs in maternal gut microbiota at the time of delivery did significantly increase the odds of presence in the infant gut at age 4–10 days for many taxa, and these included some lean-associated taxa.

**Conclusions:**

Our results show differences in maternal gut microbiota composition at the time of delivery by pre-pregnancy weight and GWG, but these changes were only associated with limited compositional differences in the early life gut microbiota of their infants. Further work is needed to determine the degree to which these maternal microbiota differences at time of birth with OW/OB and GWG may affect the health of the infant over time and by what mechanism.

**Electronic supplementary material:**

The online version of this article (10.1186/s40168-017-0332-0) contains supplementary material, which is available to authorized users.

## Background

Obesity prevalence among children and adolescents has increased dramatically in recent years [1, 2]. Maternal obesity and excessive gestational weight gain (GWG) are associated with increased risk for offspring obesity, and these associations are not fully explained by genetic and lifestyle factors. Recent research suggests that the gut microbiota may also contribute towards the development of obesity, and it has been hypothesized that the gut microbiota may be a mechanism to explain the transgenerational transmission of obesity risk [3]. The maternal gut microbiota may influence infant obesity risk through in utero programming effects [4] or through vertical transfer of obesogenic gut microbiota from mother to child during birth [5] and in breastmilk [6]. Understanding the relationship between the gut microbiota and obesity in mothers and their children may offer unique opportunities to interrupt the cycle of obesity [3].

There is increasing evidence to support that the gut microbiota may play a causal role in obesity [7, 8]. Some studies have shown differences in maternal gut microbiota at different timepoints during pregnancy by obesity status and gestational weight gain [9, 10]. Research about whether these maternal characteristics are associated with differences in the early infant gut microbiota has been inconsistent [11–13]. However, gut microbiota during infancy has been associated with rapid early growth and later overweight and obesity [14, 15], and numerous exposures known to impact the early gut microbiota, such as birth via cesarean section and antibiotics, have also been associated with increased obesity risk [16, 17].

In this study, we evaluate whether maternal pre-pregnancy overweight/obese (OW/OB) or excessive gestational weight gain (GWG) are associated with differences in the maternal gut microbiota at the time of delivery or in the gut microbiota of their infants during the first 2 years of life. We also assess the similarity between maternal gut microbiota at the time of delivery and early infant gut microbiota. This is a very important area of research because the gut microbiota is alterable—through diet, pre- and pro-biotics, and antibiotic usage. Understanding the relationship between the gut microbiota and obesity in mothers and their infants may offer opportunities for obesity prevention measures.

## Methods

### Study cohort

NoMIC is a Norwegian birth cohort of 552 children designed to study the establishment of gut microbiota during infancy and its consequences for child health. Participating mothers, recruited between 2002 and 2005, were asked to fill out periodic questionnaires and to collect and freeze fecal samples from themselves at 4 days post-partum, and from their infants at days 4, 10, 30, 120, 365, and 730 post-birth. Study personnel retrieved the fecal samples and kept them frozen during transport to the Biobank of the Norwegian Institute of Public Health, Oslo, where they were stored at −20 °C upon arrival.

The study was approved by the Regional Ethics Committee for Medical Research in Norway (approval ref. 2002, S-02216) and the Norwegian Data Inspectorate (ref 2002/1934–2). The approvals, as well as informed consent from the mothers, were obtained prior to collection of data and samples. The NoMIC study was funded by the FRIMEDBIO program at the Norwegian Research Council.

### Study sample

This study includes the subset of 169 mothers from NoMIC who provided a fecal sample, whose sample provided high quality Illumina data, and for whom both height and pre-pregnancy weight were available to calculate BMI (Additional file 1: Figure S1). Additionally, 844 gut microbiota samples during the first 2 years of life were available from the 181 children of these women (Additional file 1: Figure S1). This study sample showed some differences from the larger NoMIC cohort; they had lower median BMI, were more educated, and were less likely to smoke during pregnancy (Additional file 1: Table S1).

### Exposure definitions

Pre-pregnancy BMI was based on maternal self-report of weight at the first clinic visit; the median time of the first visit was at 9 weeks of gestation (IQR 7.3–11.3 weeks). At that visit, height and weight were measured and information on pre-pregnancy weight was obtained from the mother. A large discrepancy within the time frame of only 9 weeks would likely have been noted by the health workers. Moreover, GWG has been validated in our study (see below). Pre-pregnancy BMI was initially categorized as underweight, normal weight, overweight, and obese according to standard definitions [18]. We then further combined these groups into the following: (1) non-OW/OB: underweight (*N* = 7) and normal weight (*N* = 110), BMI < 25 (*N* = 117); and (2) OW/OB: overweight (*N* = 32) and obese (*N* = 20), BMI > 25 (*N* = 52).

When evaluating the impacts of excessive GWG on the gut microbiota, women who were missing GWG (*N* = 1) and not full term (*N* = 50) were excluded because there are not well-established weight gain recommendations for pre-term births. Furthermore, we chose to exclude mothers of twins since there were only two, making this difficult to account for in statistical models, and since the weight gain guidelines for mothers of twins are considered provisional. Thus, the sample size for the analysis of GWG was *N* = 116 (Additional file 1: Figure S1). The recommended range of the Institute of Medicine (IOM) were used to define adequate GWG, which is based on pre-pregnancy BMI (Additional file 1: Table S2); weight gain less than the recommended range for the respective BMI group was considered “low” (*N* = 12), within the range as “adequate” (*N* = 41) and greater was considered “excessive” (*N* = 63) [19]. We grouped low and adequate (*N* = 41) GWG together due to the small number in the low group, and compared to excessive GWG. GWG was calculated using the pre-pregnancy weight and final weight from self-report in a questionnaire approximately 1-month post-delivery. When missing self-reported final weight (9%), the final recorded weight preceding birth in the medical records of prenatal clinic visits was used. Self-reported GWG has been validated in the larger NoMIC study among 380 subjects with both self-reported and objective records of GWG obtained from their pregnancy journals. The Spearman correlation between the self-reported GWG and GWG according to pregnancy journals was 0.95, with a *p* value of < 0.001 and the corresponding intraclass correlation (ICC) was 0.94, with a *p* value of < 0.001.

### Additional data sources

Maternal questionnaires provided information on mode of delivery, education, parity, maternal smoking, ethnicity, and use of antibiotics. Maternal age at delivery was calculated based on birth date from the Norwegian personal identification number. We obtained information on gestational age and preterm delivery from the Medical Birth Registry of Norway.

### Processing of microbial samples

DNA was extracted using standard protocols, as previously described for this cohort [20]. The extracted DNA was amplified using polymerase chain reaction (PCR) with barcoded primers targeting the V4 region of 16S ribosomal RNA (rRNA). Sequences were generated using an Illumina HiSeq instrument (Illumina, San Diego, CA). Operational taxonomic units (OTUs) were assigned using UCLUST [21] as implemented in QIIME [22] via a closed reference-based system using the Greengenes 13.8 [23] database and a 97% threshold. A rarefied OTU table at 5000 sequences per sample served as input for the analyses.

### Statistical analysis

We compared maternal demographic and birth characteristics by pre-pregnancy weight group and excessive GWG status using chi-squared tests for categorical variables and Wilcoxon rank-sum tests for continuous variables.

#### Maternal alpha diversity

Alpha diversity measures the microbial diversity of each sample. There are many alpha diversity measures, and they differ in how they weight richness and evenness and whether they incorporate phylogenetic distance. We chose to evaluate three measures of alpha diversity: Shannon diversity index (evenly weights richness and evenness), PD whole tree (emphasizes phylogenetic diversity), and observed species (number of OTUs observed at standardized sequencing depth; richness). Rank-based regression was used to model alpha diversity measures with pre-pregnancy weight status as the primary covariate of interest. The following maternal characteristics were controlled for in the analysis: maternal age, education (< 12 years, 12 years, > 12 years), Norwegian ethnicity, parity (nulliparous, 1 prior child, > 1 prior child), twins, and smoking during pregnancy (never, former, occasional, daily ≤ 10, daily > 10). The same methods were utilized for comparing women by GWG group, except that twins were excluded from the list of covariates since all births were singleton.

#### Association between maternal characteristics and maternal microbiota composition

We visualized relationships between microbiota diversity across samples using principal coordinate analysis (PCoA) plots of weighted and unweighted UniFrac distance [24]. Random forests were first used as a feature selection technique in order to evaluate which OTUs were most important to differentiate samples based on maternal pre-pregnancy OW/OB status [25]. This machine learning approach ranks factors in terms of their ability to discriminate exposure status, while taking into account the interrelationships in high-dimensional complex data. This method uses decision trees, and each tree is trained on a subset of the data and then tested on the remaining data; the error of these repeated tests is called the “out of bag” error rate. Breiman’s random forest algorithm with down sampling [25] was used to classify women’s pre-pregnancy weight group based on the percent abundance of the OTUs. All OTUs that were present in less than 10% of the samples and those with a maximum percent abundance less than 0.25% were excluded; 448 OTUs met these prevalence cutoffs. The R function varselRF [26] was used to select the most important taxa for classifying the exposure. This is a recursive technique that eliminates variables based on the importance scores. We evaluated the classification accuracy of these selected features by computing the ratio of the out of bag error from a random forest using these features to classify simulated random data to the error from a similar random forest classifying the true exposure status [27].

Since random forests do not provide information on the nature of the relationships between these exposures and the selected taxa, we assessed the direction of the associations by modeling the OTUs individually as outcomes in beta-binomial generalized linear regressions, which account for overdispersion in the sequence counts, using the SAS procedure NLMIXED. The models included the total number of sequences (5000 for all taxa) as an offset in order to allow for inference on the relative abundance. We also included covariates in the models, controlling for the same maternal characteristics as in the models of alpha diversity.

These methods were repeated for different scenarios. First, genus level taxonomies were used rather than OTUs to model pre-pregnancy weight group with the same prevalence cutoffs as for OTUs, leaving 169 genera. We used these same methods to model excess GWG in the subset of women who were full term with singleton births, both at the OTU level and the genus level of taxonomy. The random forest of OTUs included the 403 OTUs that met the prevalence cutoffs; the random forest at the genus level included 80 genera.

We also performed sensitivity analyses. First, we examined continuous exposures of maternal pre-pregnancy BMI rather than OW/OB, and GWG (kg) in excess of the recommended GWG range rather than the dichotomous measure. Both of these showed fairly consistent taxonomic associations with the gut microbiota. We also excluded overweight women, comparing OB to non-overweight/obese. However, the classification accuracy was worse than in the primary analysis.

#### Association between maternal characteristics and infant microbiota alpha diversity and composition over the first 2 years of life

In order to evaluate the association between exposure to maternal OW/OB or excessive GWG and infant gut alpha diversity, we used longitudinal hierarchical linear regressions with a random intercept for infant within family. We controlled for the same maternal characteristics as in the models of maternal alpha diversity, in addition to the following potential mediating variables: delivery mode at birth (cesarean section or vaginal), gestational age at birth, exclusive breastfeeding (yes/no at the time of the sample), and antibiotic exposure (yes/no at the time of the sample).

Random forests were used to identify which OTUs in infant gut microbiota samples over the first 2 years of life were most important to differentiate samples based on (1) maternal OW/OB and (2) excessive GWG [25]. The same prevalence thresholds and methods were used as in the random forests of maternal gut microbiota samples, as described above. In addition to the OTUs, we included sampling time as a predictor, to allow for interactions between taxonomic abundance and age. The analysis of maternal OW/OB included 253 OTUS; the analysis of GWG included 251 OTUs.

#### Comparison of maternal and early infant gut microbiota

To compare maternal gut microbiota taxa at the time of delivery with that of the infant at days 4 and 10, we used longitudinal binary logistic regression with a random intercept by child. OTU-level presence/absence of maternal samples was used as the exposure with infant presence/absence as the outcome for each of the OTUs selected to differentiate maternal OW/OB status or excessive GWG. A similar logistic model was used to evaluate presence/absence across all OTUs together, as well as across all of OTUs selected to differentiate the maternal exposures, in order to assess the overall maternal-infant taxonomic association.

We used SAS v9.4 (SAS Institute Inc., Cary, North Carolina), R v3.2.0 [28], and QIIME v1.9.0 [22] for analyses. *p* values less than 0.05 were considered statistically significant.

## Results

Women with and without excess pre-pregnancy weight generally had similar characteristics (Table 1). Excess weight women had slightly less education and were more likely to be smokers, as were women with excessive GWG (Additional file 1: Table S3). There was a higher proportion of women who were overweight pre-pregnancy among those with excessive GWG relative to those with non-excessive GWG.

**Table 1:** Demographic and birth characteristics of mothers by pre-pregnancy weight group: overweight/obese (OW/OB) and non-OW/OB

Variable	Total (*N* = 169)	Non-OW/OB (*N* = 117)	OW/OB (*N* = 52)	*p* value
	*N* (%) or median (IQR)			
BMI	23.1 (21.1–25.5)	21.6 (20.2–23.1)	27.2 (25.7–31.7)	<.001
Pre-pregnancy BMI category				
Underweight	7 (4.1%)	7 (6.0%)	0 (0.0%)	<.001
Normal	110 (65.1%)	110 (94.0%)	0 (0.0%)	
Overweight	32 (18.9%)	0 (0.0%)	32 (61.5%)	
Obese	20 (11.8%)	0 (0.0%)	20 (38.5%)	
Ethnic Norwegian	142 (87.1%)	95 (84.1%)	47 (94.0%)	0.13
Maternal education				
<12 years education	9 (5.6%)	4 (3.6%)	5 (10.4%)	0.01
12 years education	28 (17.5%)	19 (17.0%)	9 (18.8%)	
>12 years education	123 (76.9%)	89 (79.5%)	34 (70.8%)	
Maternal age (years)	30.0 (27.0–34.0)	30.0 (27.0–33.0)	30.0 (27.5–34.0)	0.90
Parity				
No prior pregnancies	83 (49.1%)	57 (48.7%)	26 (50.0%)	0.62
1 prior child	53 (31.4%)	39 (33.3%)	14 (26.9%)	
>1 prior child	33 (19.5%)	21 (17.9%)	12 (23.1%)	
Twins	11 (6.5%)	8 (6.8%)	3 (5.8%)	1.00
Maternal smoking at beginning of pregnancy				
Never smoker	111 (66.5%)	77 (67.0%)	34 (65.4%)	0.01
Past smoker	41 (24.6%)	28 (24.3%)	13 (25.0%)	
Occasional	7 (4.2%)	5 (4.3%)	2 (3.8%)	
Daily smoker ≤10	4 (2.4%)	2 (1.7%)	2 (3.8%)	
Daily smoker >10	4 (2.4%)	3 (2.6%)	1 (1.9%)	
Diabetes				
No diabetes	167 (98.8%)	116 (99.1%)	51 (98.1%)	0.21
T1	1 (0.6%)	1 (0.9%)	0 (0.0%)	
T2	1 (0.6%)	0 (0.0%)	1 (1.9%)	
Glucose in urine	15 (8.9%)	8 (6.8%)	7 (13.5%)	0.16
High BP	8 (4.7%)	4 (3.4%)	4 (7.7%)	0.25
Gestational weight gain (kg)	14.0 (10.0–18.0)	15.0 (12.0–19.0)	11.0 (8.1–15.0)	<.001
GWG relative to IOM recommendations				
Low	12 (10.3%)	12 (14.6%)	0 (0.0%)	0.06
Adequate	41 (35.3%)	27 (32.9%)	14 (41.2%)	
Excessive	63 (54.3%)	43 (52.4%)	20 (58.8%)	
Gestational age (days)	278 (254–285)	279 (256–285)	277 (253–287)	0.80
Birth weight (kg)	3.37 (2.46–3.75)	3.31 (2.47–3.69)	3.44 (2.46–3.84)	0.29
C-section	50 (29.6%)	31 (26.5%)	19 (36.5%)	0.19
Maternal antibiotics	52 (32.1%)	35 (31.0%)	17 (34.7%)	0.71
Day of birth	9 (5.6%)	6 (5.3%)	3 (6.1%)	1.00
Day before birth	2 (1.2%)	1 (0.9%)	1 (2.0%)	0.51
Week before birth	6 (3.7%)	2 (1.8%)	4 (8.2%)	0.07
Month before birth	9 (5.6%)	6 (5.3%)	3 (6.1%)	1.00
>1 month before birth	26 (16.0%)	18 (15.9%)	8 (16.3%)	1.00
Antibiotics given to newborn	14 (8.6%)	7 (6.2%)	7 (14.3%)	0.13
Alpha diversity				
Shannon	5.7 (5.3–6.1)	5.7 (5.4–6.1)	5.5 (5.0–5.9)	0.05
Observed species	365 (307–400)	375 (315–400)	338 (292.5–385)	0.04
PD whole tree	27.3 (23.6–30.1)	28.5 (24.0–30.6)	25.9 (21.8–29.4)	0.04

### Maternal alpha diversity

The median alpha diversity was significantly lower among OW/OB women for all three alpha diversity measures examined (Table 1). In regression models controlling for maternal age, education, Norwegian ethnicity, parity, twins, and smoking during pregnancy (Table 2), Shannon diversity and PD whole tree remained significantly lower among OW/OB women. Controlling for gestational age and maternal antibiotic use during pregnancy did not substantially alter the results.

**Table 2:** Results of rank-based regression models of alpha diversity of maternal gut microbiota samples at the time of delivery

	Shannon		PD		Observed species	
	*β* (95% CI)	*p* value	*β* (95% CI)	*p* value	*β* (95% CI)	*p* value
Intercept	5.82 (5.01, 6.62)	<0.01	28.09 (21.03, 35.15)	<0.01	353.41 (262.22, 444.6)	<0.01
Maternal OW/OB	−0.19 (−0.38, −0.01)	0.04	−1.74 (−3.36, −0.12)	0.04	−19.85 (−40.52, 0.83)	0.06
Maternal age	0.02 (−0.01, 0.04)	0.14	0.09 (−0.12, 0.29)	0.40	1.79 (−0.82, 4.41)	0.18
Norwegian	−0.27 (−0.53, −0.01)	0.04	−1.45 (−3.73, 0.82)	0.21	−32.73 (−61.78, −3.69)	0.03
Education	−0.11 (−0.27, 0.05)	0.20	−0.33 (−1.74, 1.07)	0.64	−1.22 (−19.16, 16.72)	0.89
Parity: >1 prior child	−0.1 (−0.37, 0.17)	0.48	−0.38 (−2.78, 2.02)	0.76	−10.59 (−41.26, 20.08)	0.50
Parity: 1 prior child	−0.15 (−0.34, 0.05)	0.14	−1.46 (−3.18, 0.26)	0.10	−24.25 (−46.19, −2.31)	0.03
Twins	−0.18 (−0.53, 0.16)	0.31	−1.54 (−4.59, 1.5)	0.32	−16.01 (−54.94, 22.91)	0.42
Maternal smoking	−0.07 (−0.17, 0.02)	0.15	−0.54 (−1.41, 0.32)	0.22	−6.08 (−17.14, 4.98)	0.28

β indicates the parameter estimate and 95% CI is the 95% confidence interval

In the subset of full term women, excessive GWG was not associated with significant differences in alpha diversity (Shannon: *β* = − 0.1, 95% CI − 0.3, 0.1; *p* value = 0.53; PD: *β* = − 0.1, 95% CI − 1.9, 1.8; *p* value = 0.96; observed species: *β* = −4.0, 95% CI − 28.3, 20.2; *p* value = 0.75). Controlling for maternal BMI, gestational age and maternal antibiotic usage did not alter the null results.

### Maternal microbiota composition

The overall microbiota composition of the maternal samples resembled that typical of healthy European/US adults, with a dominance of taxa from the phyla of Firmicutes, Bacteroidetes, and Actinobacteria, and infant samples became more similar to maternal samples with age (Additional file 1: Figure S2) [29, 30]. Principal coordinate analysis plots of UniFrac distance also showed a progression of infant microbiota to one resembling maternal (adult-like) compositions over the first year of life, but did not show strong differentiation between gut microbiota samples by maternal OW/OB or excessive GWG status (Additional file 1: Figure S3).

Gut microbiota composition showed numerous differences by pre-pregnancy weight group. Using the most important OTUs from the random forest to classify pre-pregnancy weight status, the out of bag error rate was 18.9%; this classification accuracy was 2.4 times better than completely random guessing.

**Fig. 1:**
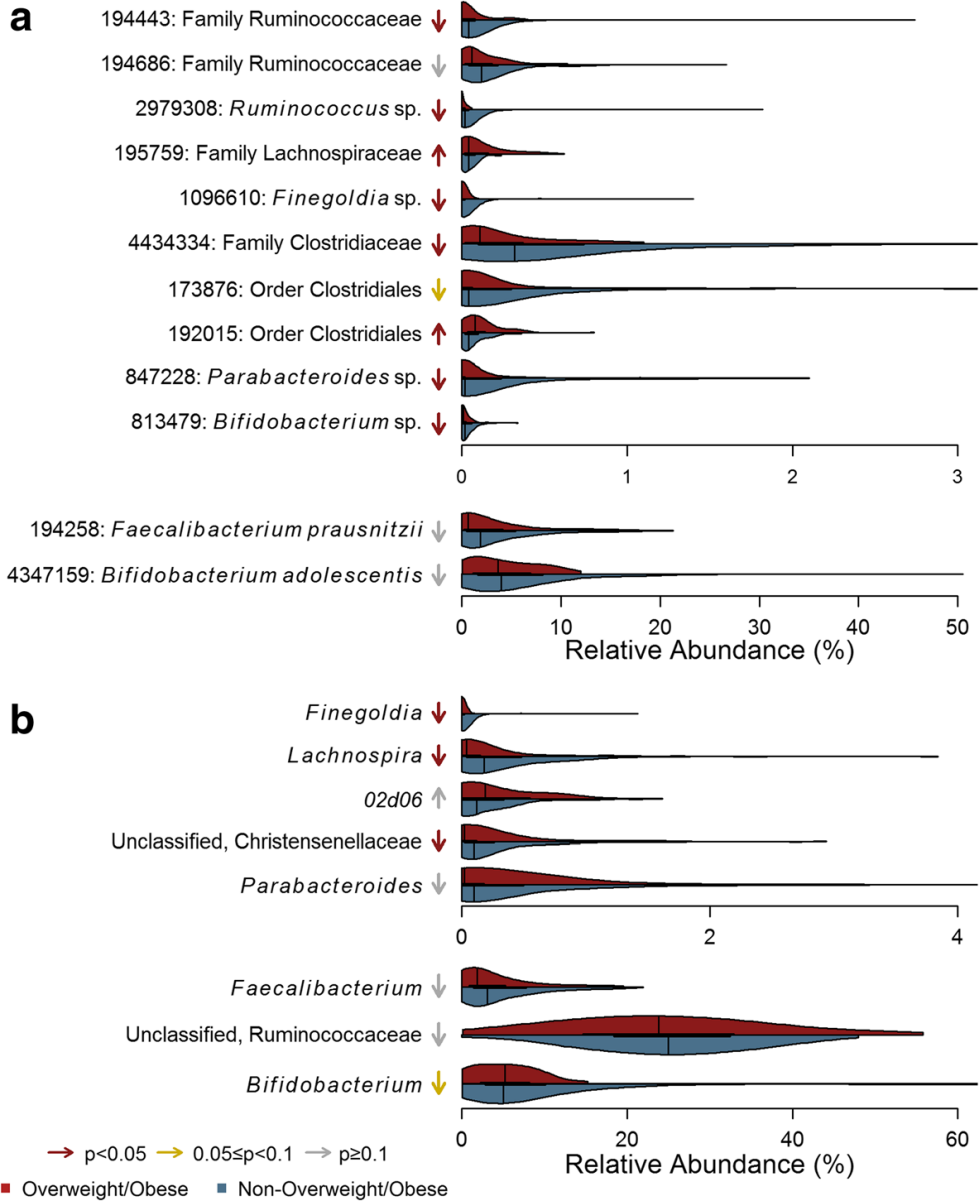
Violin plots of important **a** OTUs and **b** genera from Random Forests for classifying maternal OW/OB status. Arrows indicate the direction of association with maternal OW/OB, with color corresponding to degree of significance of uncorrected *p* values in beta-binomial regression models controlling for the following maternal characteristics: maternal age, maternal education, Norwegian ethnicity, twins, maternal smoking, and parity

The most important OTUs from the random forest included members of the genera *Bifidobacterium* and *Parabacteroides*, as well as nine members of the order Clostridiales. These taxa were modeled using beta-binomial regression models (Fig. 1; Additional file 1: Table S4). Eight of these OTUs were significantly different by maternal pre-pregnancy weight group when adjusting for maternal characteristics.

The random forest using genus level taxa to predict pre-pregnancy weight status had an out of bag error rate of 28.4%; the predictive accuracy was 1.9 times as good as random guessing. Figure 1 shows the distributions of the genus-level taxa ranked as most important in the random forest and modeled using beta-binomial regressions (Additional file 1: Table S5), which were generally consistent with the OTU-level analysis.

**Fig. 2:**
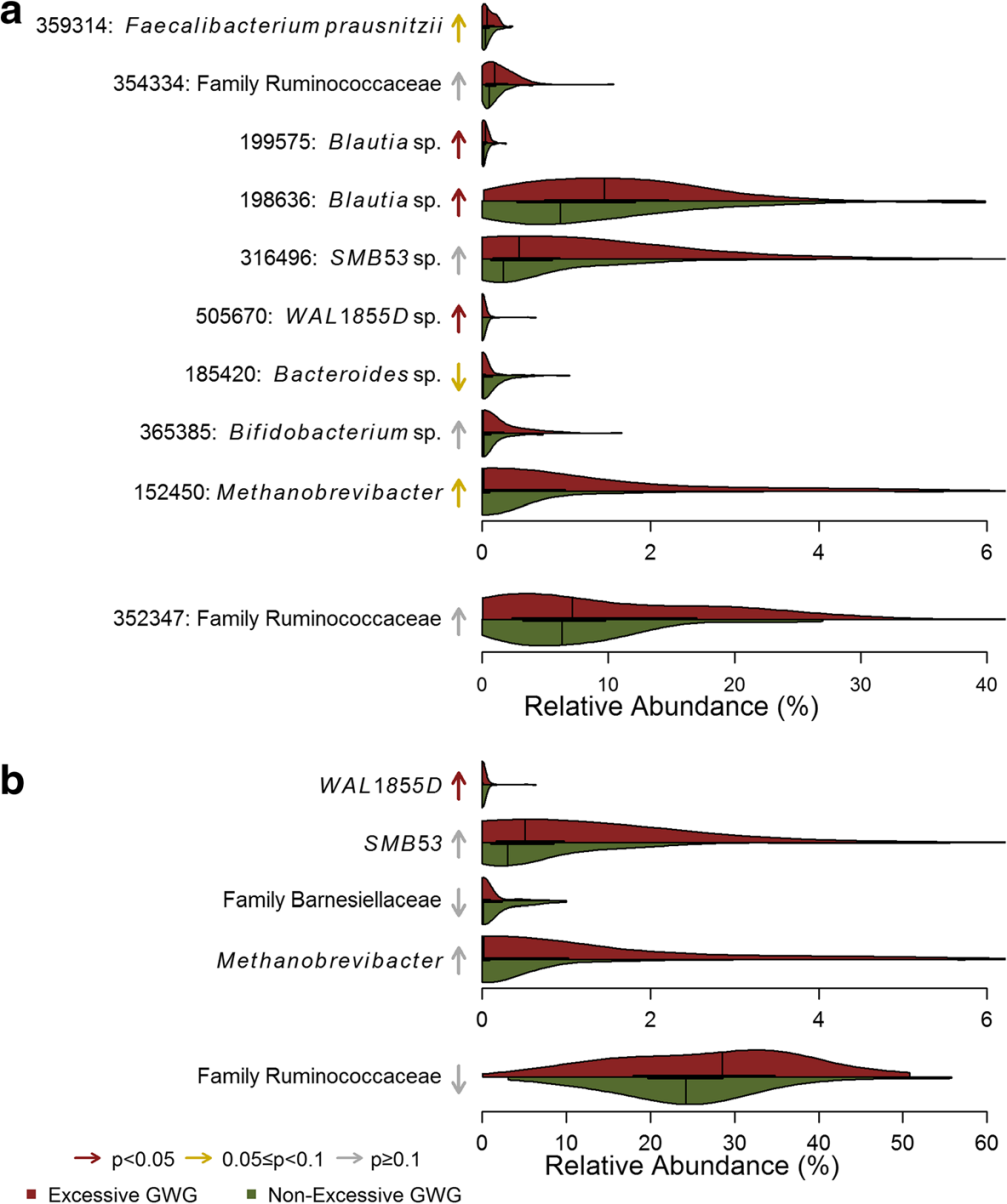
Violin plots of important **a** OTUs and **b** genera from Random Forests for classifying maternal excessive GWG status. Arrows indicate the direction of association with maternal excessive GWG, with color corresponding to degree of significance of uncorrected *p* values in beta-binomial regression models controlling for the following maternal characteristics: maternal age, maternal education, Norwegian ethnicity, maternal smoking, and parity

The random forest to classify GWG group based on OTUs had an out of bag error rate of 24.1%; this classification accuracy was twice as accurate as random guessing. The most important taxa included members of the genera *Methanobrevibacter*, *Bifidobacterium*, and *Bacteroides*, as well as seven OTUs of the order Clostridiales. In the beta-binomial regression models, there were three OTUs that were significantly higher among women with excess GWG when controlling for maternal characteristics (Fig. 2; Additional file 1: Table S6). The random forest using genus-level taxa as predictors had an out of bag error rate of 31.9%, which was 1.7 times as good as random guessing, and showed consistent patterns as the OTU level analysis (Fig. 2; Additional file 1: Table S7).

### Association between maternal characteristics and infant microbiota alpha diversity and composition over the first 2 years of life

The alpha diversity of infant gut microbiota over the first 2 years of life did not show an association with exposure to maternal OW/OB or excessive GWG in any of the examined measures of alpha diversity (Additional file 1: Table S8). Random forests trained to classify the infant gut microbiota according to these maternal characteristics had poor accuracy (Additional file 1: Tables S9-S10), being only 22% better than random guessing for classifying taxa by maternal OW/OB and 8% better for classifying excessive GWG. This low level of accuracy suggests that the models could not identify clinically meaningful taxonomic differences [31]. We also investigated whether other factors that have been previously associated with differences in the infant gut microbiota were likewise associated in this cohort, including mode of delivery, breastfeeding, and antibiotic exposure using a permutational ANOVA of unweighted and weighted UniFrac distance. All of these exposures were highly significantly associated (*p* values ≤ 0.002) with both the phylogeny (unweighted UniFrac) and abundance (weighted UniFrac) of the infant gut microbiota.

### Comparison of maternal and early infant gut microbiota

**Fig. 3:**
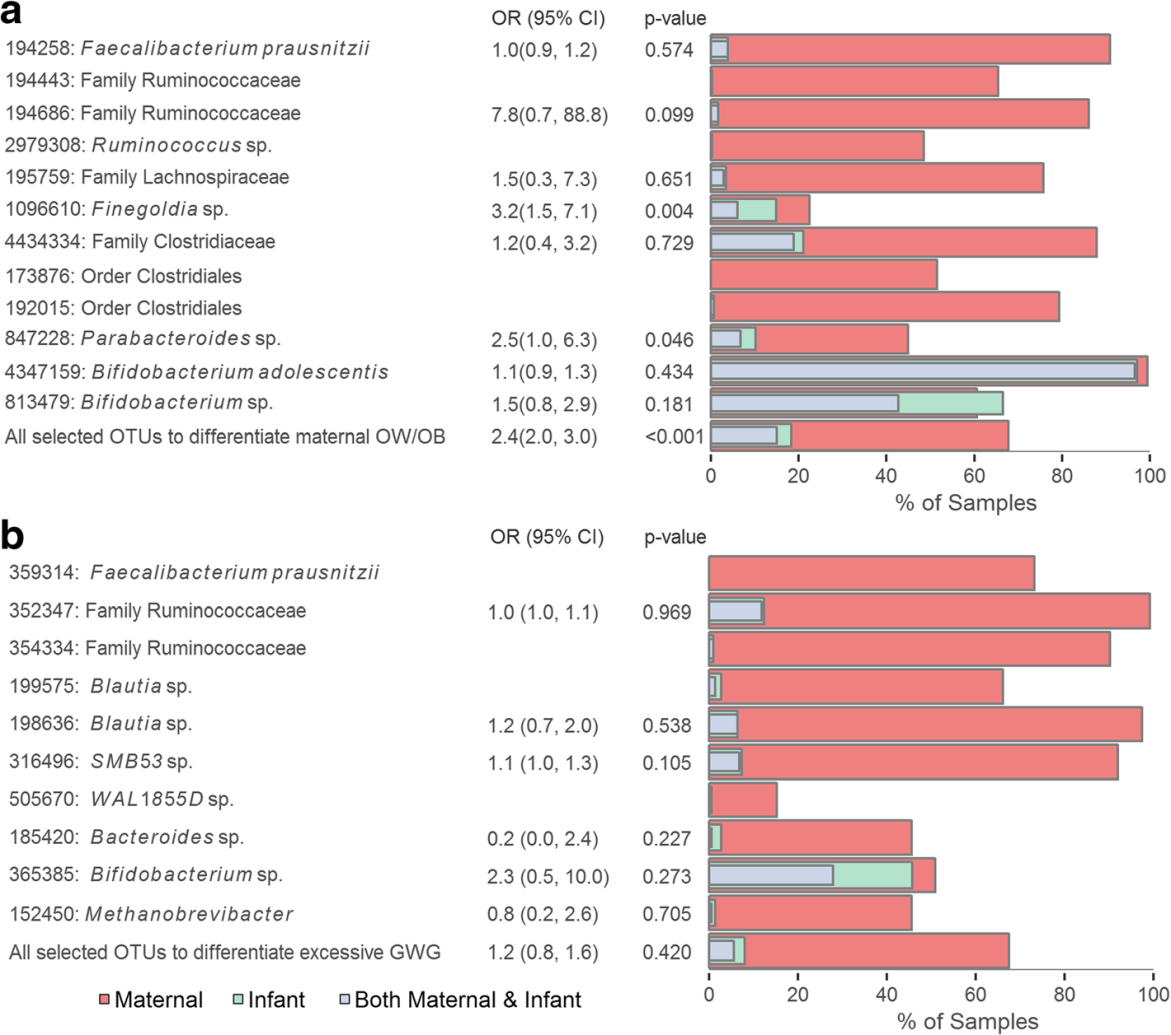
Plots of the presence of OTUs that differentiate **a** pre-pregnancy maternal OW/OB status and **b** excessive GWG in maternal and infant gut microbiota samples. The percent of samples with non-zero abundance from the mothers at the time of delivery are shown in red, and the percent of such samples from the infants at days 4–10 of life are shown in green, and from both mother and child are shown in gray for each OTU, as well as the average across these OTUs. The results of logistic regressions estimating the odds of presence in the infant based on presence in the mother are also shown. OR indicates the odds ratio, and 95% CI is the 95% confidence interval. In some cases, there was not enough data or enough variation in the presence of the OTUs across the samples to estimate the regression parameters

The presence of OTUs in maternal gut microbiota at the time of delivery significantly increased the odds of presence in the early infant gut microbiota; this was true when evaluating the average association across all OTUs (OR = 2.8, 95% CI 2.8, 2.9; *p* value < 0.01) and when specifically evaluating those associated with maternal OW/OB (Fig. 3; OR = 2.4, 95% CI 2.0, 3.0; *p* value < 0.001). Yet, most of the individual OTUs associated with maternal OW/OB were not significantly associated between maternal-infant samples, and some were almost entirely absent from the infants. However, the presence of lean-associated OTUs in the *Parabacteroides* and *Finegoldia* genera in maternal samples were associated with increased odds of presence in the infant (OR = 2.5, 95% CI 1, 6.3; *p* value < 0.05; OR = 3,2, 95% CI 1.5, 7.1 *p* value < 0.01, respectively). The group of OTUs highlighted as important to classify maternal excessive GWG were not significantly associated overall between maternal and infant samples (Fig. 3; OR = 1.2, 95% CI 0.8, 1.6; *p* value = 0.42).

## Discussion

Both maternal OW/OB and excessive GWG have detrimental short- and long-term health consequences for the infant, such as increased risk for fetal macrosomia, obesity, metabolic syndrome, and even all-cause mortality [3]. It is possible that the maternal gut microbiota may mediate some of the increased disease risk associated with these exposures, particularly with respect to obesity. Our results showing numerous differences in maternal gut microbiota associated with pre-pregnancy OW/OB and GWG lend support to this notion. We found that pre-pregnancy OW/OB is associated with both lower maternal alpha diversity as well as differences in microbial composition, and that excess GWG is associated with compositional differences. While we might expect that these differences in maternal gut microbiota would translate into overall differences in their infants’ gut microbiota, we did not find that these maternal characteristics were associated with significant differences in the infant gut microbiota over the first 2 years of life. However, we did find significant correlation between presence of taxa in the maternal and infant samples, particularly for the maternal taxa associated with pre-pregnancy weight.

Most of the taxa that differentiated maternal OW/OB in our study were higher among lean women, and many of these have shown consistent associations with leanness in prior studies, such as *Parabacteroides* [32, 33], *Lachnospira* [34], *Faecalibacterium prausnitzii* [35], members of the family Christensenellaceae [36], *Ruminococcus* [37], and *Bifidobacterium* [38]. Furthermore, some of these taxa may be of particular importance in the early infant gut. Abundance of *Lachnospira* [33, 34, 39–41] and *Faecalibacterium* in the first 3 months of life have been associated with risk for developing asthma [42]. Furthermore, members of Christensenellaceae family are among most heritable taxa and have shown a protective effect against weight gain in mouse studies involving fecal-transplants from obese humans [36], which could make it of key importance to explain the obesity associations across generations, as well as a key target for microbiota-based interventions.

Our results show an association between maternal OW/OB and low alpha diversity, which has also been associated with obesity in some prior studies [39, 43] as well as many other diseases (including inflammatory bowel disease [35], autism [44], asthma/allergy [40], and dyslipidemia [43]) but may be a consequence of disease, as longitudinal studies are limited. Koren et al. [41] showed a decrease in alpha diversity from the first to third trimester in pregnant women, although not replicated in another study of pregnant women by DiGuilo et al. [45]

The taxa associated with excessive GWG in our results are generally distinct from those associated with pre-pregnancy OW/OB, and show less consistent patterns with prior studies of obesity. One exception is the taxa *Blautia*, which was enriched with excessive GWG and has been associated with type 2 diabetes [46], and some species of this genus have been associated with obesity in a Japanese population [47]. However, other research has suggested that *Blautia* may also be beneficial to health in some contexts [48]. The taxa that differentiated maternal excessive GWG also tended to be absent or at non-detectible levels in most of their infants at days 4–10; they tend to be later colonizers of the infant gut.

We found that maternal OW/OB and excessive GWG were not significant determinants of the infant gut microbiota composition during the first 2 years of life. This is not entirely surprising; infant gut microbiota changes drastically over the first few years of life, differs substantially across infants, and is affected strongly by other factors, including mode of delivery, breastfeeding, and antibiotic usage [5, 49]. Other studies have also examined these associations, and the results have been inconsistent. Collado et al. found numerous differences according to maternal pre-pregnancy weight and gestational weight gain (GWG) in infant samples at 1 and 6 months [50]; Mueller et al. found that microbiota from the first infant stool differed by maternal OW/OB *only* among vaginally born infants [11]; Galley et al. found differences at 18–27 months *only* among infants of high socioeconomic status [13]; and Laursen et al. found no impact of maternal obesity on microbial diversity or taxonomic composition at 9 or 18 months [12]. This prior research combined with our results suggest that maternal pre-pregnancy weight and GWG are not major determinants of the overall taxonomic composition of the infant gut. However, it is possible that these maternal characteristics may influence specific infant gut microbes that affect later obesity risk. It is also possible that the effect of these characteristics is more pronounced among infants with the most exposure to maternal gut microbiota—e.g., those who are vaginally born, breastfed, and unexposed to antibiotics in early life. We aim to explore this hypothesis in future work in the full cohort of NoMIC infants.

The differences noted in the maternal microbiota could potentially influence the offspring through various means, including vertical transfer during and after birth which then shapes the gut colonization process of the infant. Work by Backhed et al. [5] showed that the maternal gut microbiota is a major determinant of the infant gut microbiota; 72% of early colonizers in the infant gut matched species found in maternal samples for vaginally born infants, and 41% in infants born via C-section. Similarly, we see that the presence of taxa in the maternal gut microbiota at the time of delivery is highly predictive of presence in the infant in early life. A recent study by Nayfach et al. [51] performed a strain-level analysis to further explore the variation in maternal-infant vertical transmission across taxa and over the first year of life. They found evidence of extensive vertical transmission of gut microbiota shortly after birth, particularly for certain species, including *Parabacteroides distasonis* and *Bifidobacterium adolescentis*, and that the vast majority of strains in the infants at 4 days that were transmitted from the mother persisted in the infants at 4 and 12 months. This is interesting in the context of our results because species in the *Parabacteroides* and *Bifidobacterium* genera were both associated with leanness in mothers, and presence of these taxa in the mother was predictive of presence in their infants.

Some of the taxa associated with maternal pre-pregnancy OW/OB or excessive GWG tend to be later colonizing bacteria, such as *Blautia*, *Ruminococcus*, and *Faecalibacterium*, and they may not be transferred from the mother. However, Nayfach’s work also showed that while strain-level similarity between mothers and infants significantly decreased over the first year of life, the maternal-infant species-level composition converged over time [51]*.* Thus, while these taxa may not be vertically transmitted to the infants, their presence in the mother may still be predictive of species-level composition in their infants at later timepoints due to shared environmental exposures, and may still impact the infant risk for obesity.

The differences in the maternal microbiota seen in our results could also have in utero impacts. The fetal programming hypothesis sets forth the notion that exposures in utero, at birth and in early life may have long-term effects on adult health [52]. A recent study by Agüero et al. [4] provides compelling evidence that in utero programming occurs, in part, through the maternal gut microbiota. Using a murine model, they found that transient maternal colonization during pregnancy had long-term effects on the innate immune system of the offspring, as well as the expression of numerous genes in the newborn intestine, including genes involved in metabolism and oxidative stress.

This study has both important strengths and limitations. Our population is much larger than many of the prior studies of maternal weight and the gut microbiota, and it includes gut microbiota data from both the mothers at the time of delivery and their infants over the first 2 years of life. The maternal vaginal and skin microbiota may play important roles in seeding the infant microbiome [53], but only gut microbiota samples were collected in this study. The population is largely of Norwegian ethnicity, which means that some of the taxonomic findings may not reflect patterns in other ethnic or racial groups, or in other geographic regions. However, the results should be internally consistent in terms of showing support for the notion that maternal weight characteristics may influence maternal gut microbiota at the time of delivery, which may affect some of the early infant taxa. Methodologically, we used machine learning techniques, which are particularly suited to the analysis of complex gut microbiota data [27], and regression models that controlled for many of the known confounders of the relationship between maternal weight and the gut microbiota. However, it is difficult to completely control for certain effects, particularly for the infant samples, which are known to be largely influenced by breastfeeding, mode of delivery, and anti-biotic exposure [5, 49].

Many of the gut microbiota associated with OW/OB in pregnant women in this study have been previously associated with obesity in non-pregnant adults. Some of the taxonomic differences noted, while not previously associated with obesity, have been associated with childhood risk for other conditions, such as asthma. Thus, pre/pro-biotics targeted towards obesity in the general population may be beneficial for pregnant women as well; however, there may be additional microbiota that are particularly advantageous for pregnant women around the time of delivery. For example, bacteria from the genera of *Parabacteroides* or *Bifidobacterium*, or those from the family Christensenellacea, may play a protective role against excessive weight gain and be highly heritable. These and other gut microbiota highlighted in this study offer insight into the etiology of childhood obesity and may inform future studies related to obesity prevention efforts based on the gut microbiota.

## Conclusions

In this study, we found that maternal OW/OB and excessive GWG were associated with differences in the maternal gut microbiota at the time of delivery. These changes were only associated with limited compositional differences in their infants. The differences seen in maternal gut microbiota could have health consequences for the child through programming effects or direct seeding of the infant gut microbiota. However, further research is needed to understand whether the maternal or infant gut microbiota are key mechanisms for the transgenerational transmission of obesity risk.

## Additional file

Additional file 1: Supplemental tables and figures. (PDF 1411 kb)

## Abbreviations

GWG: Gestational weight gain
OTU: Operational taxonomic unit
OW/OB: Overweight/obese
