# Supplementary material for: Pre-pregnancy weight, gestational weight gain, and the gut microbiota of mothers and their infants
Source: Microbiome. 2017 Sep 4;5:113. doi: 10.1186/s40168-017-0332-0 (PMC5584478; doi:10.1186/s40168-017-0332-0)
Supplement: Additional file 1: — Supplemental tables and figures. (PDF 1411 kb) [file 40168_2017_332_MOESM1_ESM.pdf]

Legends

Supplemental Tables

Table S1. Demographic and birth characteristics of mothers in NoMIC with non-missing pre-pregnancy BMI information by inclusion in the study sample (i.e. with viable gut microbiota samples).

Table S2. Institute of Medicine's recommended range of adequate gestational weight gain based on pre-pregnancy BMI; weight gain less than the recommended range was considered “low” and greater was considered “excessive.”

Table S3. Demographic and birth characteristics of mothers by GWG group: excessive and non-excessive GWG

Table S4. Results of Beta-Binomial Models for the most important OTUs from random forests (RF) to classify women based on pre-pregnancy weight group. Taxonomy indicates the green genes taxonomy. Adjusted p-values account for the following maternal characteristics: maternal age, maternal education, Norwegian ethnicity, twins, maternal smoking, and parity.

Table S5. Results of Beta-Binomial Models for most important genera from random forests (RF) to classify women based on pre-pregnancy weight group. Adjusted p-values account for the following maternal characteristics: maternal age, maternal education, Norwegian ethnicity, twins, maternal smoking, and parity.

Table S6. Results of Beta-Binomial Models for the most important OTUs from random forests (RF) to classify women based on GWG group. Taxonomy indicates the green genes taxonomy. Adjusted p-values account for the following maternal characteristics: maternal age, maternal education, Norwegian ethnicity, maternal smoking, and parity.

Table S7. Results of Beta-Binomial Models for the most important genera from random forests (RF) to classify women based on GWG group. Adjusted p-values account for the following maternal characteristics: maternal age, maternal education, Norwegian ethnicity, maternal smoking, and parity.

Table S8. Association between exposure to Maternal OW/OB and 3 measures of infant alpha diversity during the first two years of life, both unadjusted and adjusted for exclusive breastfeeding, mode of delivery at birth, antibiotic exposure, and gestational age at birth. Results are estimates from longitudinal hierarchical linear regression models.

Table S9. Results of random forest selection process to identify infant gut microbiota taxa associated with exposure to maternal OW/OB, sorted from most to least important according to the Mean Decrease in Accuracy. The out of bag error was 32.9%. For a similar random forest classifying randomly assigned exposure status, the out of bag error was only 21.6% better, indicating that the model failed to identify significant taxonomic differences with exposure.

Table S10. Results of random forest selection process to identify infant gut microbiota taxa associated with exposure to excessive GWG, sorted from most to least important according to the Mean Decrease in Accuracy. The out of bag error was 36%. For a similar random forest classifying randomly assigned exposure status, the out of bag error was only 8% better, indicating that the model failed to identify significant taxonomic differences with exposure.

Supplemental Figures

Figure S1. Flow chart for the samples from the NoMIC cohort included in this study.

Figure S2. Most abundant a) phylum and b) genus level taxa in maternal samples at the time of delivery and infant samples at days 4, 10, 30, 120, 365, and 730 post-birth by maternal pre-pregnancy overweight / obese (OW/OB) status.

Figure S3. Principal coordinate analysis plots of weighted (left) and unweighted (right) UniFrac distance of maternal gut microbiota samples 4 days post-partum and infant gut samples at days 4, 10, 30, 120, 365, and 730 post-birth by a) sampling time, b) maternal overweight / obesity (OW/OB) and c) maternal excessive gestational weight gain (GWG).

Supplemental Tables

Table S1. Demographic and birth characteristics of mothers in NoMIC with non-missing pre-pregnancy BMI information by inclusion in the study sample (i.e. with viable gut microbiota samples).

**Variable**	**Total (N=169)**	**Not in Study Sample (N=309)**	**In Study Sample (N=169)**	**P-Value**
	N (%) or Median (IQR)			
BMI	23.9 (21.3-27.1)	24.7 (21.5-27.7)	23.1 (21.1-25.5)	0.01
Pre-pregnancy BMI category				
Underweight	24 (5.0%)	17 (5.5%)	7 (4.1%)	0.01
Normal	261 (54.6%)	151 (48.9%)	110 (65.1%)	
Overweight	128 (26.8%)	96 (31.1%)	32 (18.9%)	
Obese	65 (13.6%)	45 (14.6%)	20 (11.8%)	
Ethnic Norwegian	388 (88.0%)	247 (88.5%)	141 (87.0%)	0.64
Maternal Education				
<12yrs education	56 (12.1%)	47 (15.6%)	9 (5.6%)	0.01
12yrs education	83 (18.0%)	55 (18.2%)	28 (17.5%)	
>12yrs education	323 (69.9%)	200 (66.2%)	123 (76.9%)	
Maternal Age (years)	30.0 (27.0-33.0)	30.0 (27.0-33.0)	30.0 (27.0-34.0)	0.35
Parity				
No prior pregnancies	207 (43.3%)	124 (40.1%)	83 (49.1%)	0.16
1 prior child	171 (35.8%)	118 (38.2%)	53 (31.4%)	
>1 prior child.	100 (20.9%)	67 (21.7%)	33 (19.5%)	
Twins	33 (6.9%)	22 (7.1%)	11 (6.5%)	0.80
Maternal Smoking at Beginning of Pregnancy				
Never smoker	275 (58.4%)	163 (53.6%)	112 (67.1%)	<0.001
Past smoker	128 (27.2%)	88 (28.9%)	40 (24.0%)	
Occasional	17 (3.6%)	10 (3.3%)	7 (4.2%)	
Daily smoker <=10	44 (9.3%)	40 (13.2%)	4 (2.4%)	
Daily smoker >10	7 (1.5%)	3 (1.0%)	4 (2.4%)	
Diabetes				
No Diabetes	467 (97.7%)	300 (97.1%)	167 (98.8%)	0.20
T1	7 (1.5%)	6 (1.9%)	1 (0.6%)	
T2	1 (0.2%)	0 (0.0%)	1 (0.6%)	
Pregnancy diabetes	3 (0.6%)	3 (1.0%)	0 (0.0%)	
Glucose in urine	53 (11.1%)	38 (12.3%)	15 (8.9%)	0.25
High BP	27 (5.6%)	19 (6.1%)	8 (4.7%)	0.52
Gestational Weight Gain (kg)	14 (10.1-18)	14 (10.1-19)	14 (10.2-18)	0.91
GWG relative to IOM recommendations				
Low	46 (13.2%)	30 (13.2%)	16 (13.3%)	0.43
Adequate	107 (30.7%)	65 (28.5%)	42 (35.0%)	
Excessive	195 (56.0%)	133 (58.3%)	62 (51.7%)	
Gestational Age (days)	278 (254-286)	278 (254-286)	278 (254-285)	0.65
Birth Weight (kg)	3.4 (2.7-3.8)	3.3 (2.8-3.9)	3.4 (2.5-3.8)	0.17
C-section	158 (33.1%)	108 (35.0%)	50 (29.6%)	0.23
Maternal Antibiotics	159 (36.3%)	107 (38.6%)	52 (32.3%)	0.18
Day of Birth	25 (5.7%)	16 (5.8%)	9 (5.6%)	0.94
Day before Birth	8 (1.8%)	6 (2.2%)	2 (1.2%)	0.72
Week before Birth	18 (4.1%)	12 (4.3%)	6 (3.7%)	0.76
Month before Birth	25 (5.7%)	16 (5.8%)	9 (5.6%)	0.94
>1 Month before Birth	92 (21.0%)	66 (23.8%)	26 (16.1%)	0.06
Antibiotics given to newborn	52 (11.6%)	38 (13.3%)	14 (8.6%)	0.13

Table S2. Institute of Medicine's recommended range of adequate gestational weight gain based on pre-pregnancy BMI; weight gain less than the recommended range was considered “low” and greater was considered “excessive.”

**Prepregnancy BMI**	**BMI (kg/m^2)**	**Recommended Weight Gain (lbs / kg)**
Underweight	<18.5	28–40 lbs / 13-18 kg
Normal weight	18.5-24.9	25–35 / 11.5-16 kg
Overweight	25.0-29.9	15–25 lbs / 7-11.5 kg
Obese	≥30.0	11–20 / 5-9 kg

Table S3. Demographic and Birth Characteristics of Mothers by GWG Group: Excessive and Non-excessive GWG

Variable	Total (N=116)	Non-Excessive GWG (N=53)	Excessive GWG (N=63)	P-Value
	N (%) or Median (IQR)			
BMI	22.9 (21.2-25.3)	22.5 (21.1-25.1)	23.1 (21.2-25.4)	0.84
Pre-pregnancy BMI category				
Underweight	5 (4.3%)	4 (7.5%)	1 (1.6%)	<.001
Normal	77 (66.4%)	35 (66.0%)	42 (66.7%)	
Overweight	25 (21.6%)	8 (15.1%)	17 (27.0%)	
Obese	9 (7.8%)	6 (11.3%)	3 (4.8%)	
Overweight / Obese	34 (29.3%)	14 (26.4%)	20 (31.7%)	0.53
Ethnic Norwegian	96 (84.2%)	47 (88.7%)	49 (80.3%)	0.30
Maternal Education				
<12yrs education	4 (3.6%)	2 (3.9%)	2 (3.3%)	0.06
12yrs education	16 (14.4%)	6 (11.8%)	10 (16.7%)	
>12yrs education	91 (82.0%)	43 (84.3%)	48 (80.0%)	
Maternal Age (years)	31.0 (28.0-34.0)	31.0 (28.0-35.0)	30.0 (27.0-33.0)	0.28
Parity				
No prior pregnancies	53 (45.7%)	19 (35.8%)	34 (54.0%)	0.12
1 prior child	41 (35.3%)	21 (39.6%)	20 (31.7%)	
>1 prior child.	22 (19.0%)	13 (24.5%)	9 (14.3%)	
Twins	11 (6.5%)	8 (6.8%)	3 (5.8%)	1
Maternal Smoking at Beginning of Pregnancy				
Never smoker	82 (71.3%)	38 (71.7%)	44 (71.0%)	0.02
Past smoker	25 (21.7%)	12 (22.6%)	13 (21.0%)	
Occasional	5 (4.3%)	2 (3.8%)	3 (4.8%)	
Daily smoker <=10	3 (2.6%)	1 (1.9%)	2 (3.2%)	
Diabetes				
No Diabetes	115 (99.1%)	53 (100.0%)	62 (98.4%)	1
T1	1 (0.9%)	0 (0.0%)	1 (1.6%)	
Glucose in urine	15 (8.9%)	8 (6.8%)	7 (13.5%)	0.16
High BP	9 (7.8%)	3 (5.7%)	6 (9.5%)	0.51
Gestational Weight Gain (kg)	15.0 (12.0-18.9)	11.7 (8.0-14.0)	18.0 (17.0-22.0)	<.001
Gestational Age (days)	284.0 (277.0-287.5)	282.0 (275.0-286.0)	284.0 (277.0-288.0)	0.21
Birth Weight (kg)	3.58 (3.31-3.87)	3.44 (3.23-3.69)	3.74 (3.38-4.0)	0.003
C-section	24 (20.7%)	9 (17.0%)	15 (23.8%)	0.37
Maternal Antibiotics	31 (27.2%)	14 (26.4%)	17 (27.9%)	1
Day of Birth	3 (2.6%)	1 (1.9%)	2 (3.3%)	1
Day before Birth	1 (0.9%)	0 (0.0%)	1 (1.6%)	1
Week before Birth	3 (2.6%)	1 (1.9%)	2 (3.3%)	1.00
Month before Birth	7 (6.1%)	1 (1.9%)	6 (9.8%)	0.12
>Month before Birth	18 (15.8%)	11 (20.8%)	7 (11.5%)	0.2
Antibiotics given to newborn	5 (4.4%)	0 (0.0%)	5 (8.2%)	0.06
Alpha Diversity				
Shannon	5.8 (5.3-6.1)	5.8 (5.2-6.2)	5.7 (5.3-6.1)	0.54
N Observed Species	371.0 (311.5-403.0)	373.0 (309.0-412.0)	369.0 (312.0-393.0)	0.82
PD Whole Tree	28.0 (23.8-30.8)	28.2 (23.8-31.0)	27.6 (23.6-30.6)	0.99

Table S4. Results of Beta-Binomial Models for the most important OTUs from random forests (RF) to classify women based on pre-pregnancy weight group. Taxonomy indicates the green genes taxonomy. Adjusted p-values account for the following maternal characteristics: maternal age, maternal education, Norwegian ethnicity, twins, maternal smoking, and parity.

OTUID	Taxonomy	Excess GWG vs Non	Estimate	P-value	Adjusted P-value	RF Rank
198636	p_Firmicutes; c_Clostridia; o_Clostridiales; f_Lachnospiraceae; g_Blautia; s_	↑	0.366	0.012	0.007	10
505670	p_Firmicutes; c_Clostridia; o_Clostridiales; f_[Tissierellaceae]; g_WAL_1855D; s_	↑	1.880	0.014	0.007	5
199575	p_Firmicutes; c_Clostridia; o_Clostridiales; f_Lachnospiraceae; g_Blautia; s_	↑	0.429	0.040	0.019	6
152450	k_Archaea;p_Euryarchaeota;c_Methanobacteria;o_Methanobacteriales;f_Methanobacteriaceae;g_Methanobrevibacter	↑	0.555	0.058	0.072	7
185420	p_Bacteroidetes; c_Bacteroidia; o_Bacteroidales; f_Bacteroidaceae; g_Bacteroides; s_	↓	-0.467	0.090	0.056	4
359314	p_Firmicutes; c_Clostridia; o_Clostridiales; f_Ruminococcaceae; g_Faecalibacterium; s_prausnitzii	↑	0.317	0.106	0.086	3
354334	p_Firmicutes; c_Clostridia; o_Clostridiales; f_Ruminococcaceae; g_; s_	↑	0.198	0.230	0.128	1
316496	p_Firmicutes; c_Clostridia; o_Clostridiales; f_Clostridiaceae; g_SMB53; s_	↑	0.094	0.601	0.346	9
352347	p_Firmicutes; c_Clostridia; o_Clostridiales; f_Ruminococcaceae; g_; s_	↑	0.072	0.657	0.545	2
365385	p_Actinobacteria; c_Actinobacteria; o_Bifidobacteriales; f_Bifidobacteriaceae; g_Bifidobacterium; s_b	↑	0.041	0.872	0.788	8

Table S52. Results of Beta-Binomial Models for most important genera from random forests (RF) to classify women based on pre-pregnancy weight group. Adjusted p-values account for the following maternal characteristics: maternal age, maternal education, Norwegian ethnicity, twins, maternal smoking, and parity.

Taxonomy	OW/OB vs Non	Estimate	P-value	Adjusted P-value	RF Rank
p_Firmicutes;c_Clostridia;o_Clostridiales;f_Lachnospiraceae;g_Lachnospira	↓	-0.625	0.001	0.001	1
p_Firmicutes;c_Clostridia;o_Clostridiales;f_Christensenellaceae;g_	↓	-0.630	0.002	0.003	4
p_Firmicutes;c_Clostridia;o_Clostridiales;f_[Tissierellaceae];g_Finegoldia	↓	-1.206	0.013	0.004	5
p_Actinobacteria;c_Actinobacteria;o_Bifidobacteriales;f_Bifidobacteriaceae;g_Bifidobacterium	↓	-0.226	0.124	0.056	2
p_Firmicutes;c_Clostridia;o_Clostridiales;f_Ruminococcaceae;g_Faecalibacterium	↓	-0.223	0.150	0.158	3
p_Bacteroidetes;c_Bacteroidia;o_Bacteroidales;f_Porphyromonadaceae;g_Parabacteroides	↓	-0.233	0.255	0.167	7
p_Firmicutes;c_Clostridia;o_Clostridiales;f_Ruminococcaceae;g_	↓	-0.088	0.487	0.382	8
p_Firmicutes;c_Clostridia;o_Clostridiales;f_Clostridiaceae;g_02d06	↑	0.069	0.720	0.471	6

Table S6. Results of Beta-Binomial Models for the most important OTUs from random forests (RF) to classify women based on GWG group. Taxonomy indicates the green genes taxonomy. Adjusted p-values account for the following maternal characteristics: maternal age, maternal education, Norwegian ethnicity, maternal smoking, and parity.

OTU	Taxonomy	OW/OB vs Non	Estimate	P-value	Adjusted P-value	RF Rank
4434334	p_Firmicutes; c_Clostridia; o_Clostridiales; f_Clostridiaceae; g_; s_	↓	-0.464	0.006	0.007	1
847228	p_Bacteroidetes; c_Bacteroidia; o_Bacteroidales; f_Porphyromonadaceae; g_Parabacteroides; s_	↓	-0.720	0.011	0.009	12
192015	p_Firmicutes; c_Clostridia; o_Clostridiales; f_; g_; s_	↑	0.388	0.013	0.003	7
1096610	p_Firmicutes; c_Clostridia; o_Clostridiales; f_[Tissierellaceae]; g_Finegoldia; s_	↓	-1.170	0.016	0.005	9
2979308	p_Firmicutes; c_Clostridia; o_Clostridiales; f_Ruminococcaceae; g_Ruminococcus; s_	↓	-0.600	0.022	0.015	3
194443	p_Firmicutes; c_Clostridia; o_Clostridiales; f_Ruminococcaceae; g_; s_	↓	-0.493	0.027	0.037	10
813479	p_Actinobacteria; c_Actinobacteria; o_Bifidobacteriales; f_Bifidobacteriaceae; g_Bifidobacterium; s_	↓	-0.468	0.030	0.023	11
195759	p_Firmicutes; c_Clostridia; o_Clostridiales; f_Lachnospiraceae; g_; s_	↑	0.319	0.060	0.022	5
173876	p_Firmicutes; c_Clostridia; o_Clostridiales; f_; g_; s_	↓	-0.347	0.140	0.086	2
194686	p_Firmicutes; c_Clostridia; o_Clostridiales; f_Ruminococcaceae; g_; s_	↓	-0.219	0.171	0.312	6
194258	p_Firmicutes; c_Clostridia; o_Clostridiales; f_Ruminococcaceae; g_Faecalibacterium; s_prausnitzii	↓	-0.219	0.192	0.228	8
4347159	p_Actinobacteria; c_Actinobacteria; o_Bifidobacteriales; f_Bifidobacteriaceae; g_Bifidobacterium; s_adolescentis	↓	-0.174	0.238	0.211	4

Table S7. Results of Beta-Binomial Models for the most important genera from random forests (RF) to classify women based on GWG group. Adjusted p-values account for the following maternal characteristics: maternal age, maternal education, Norwegian ethnicity, maternal smoking, and parity.

Taxonomy	Excess GWG vs Non	Estimate	P-value	Adjusted P-value	RF Rank
p_Firmicutes;c_Clostridia;o_Clostridiales;f_[Tissierellaceae];g_WAL_1855D	↑	1.999	0.009	0.004	3
k_Archaea;p_Euryarchaeota;c_Methanobacteria;o_Methanobacteriales;f_Methanobacteriaceae;g_Methanobrevibacter	↑	0.556	0.058	0.071	2
p_Bacteroidetes;c_Bacteroidia;o_Bacteroidales;f_[Barnesiellaceae];g_	↓	-0.358	0.185	0.126	4
p_Firmicutes;c_Clostridia;o_Clostridiales;f_Clostridiaceae;g_SMB53	↑	0.133	0.450	0.264	5
p_Firmicutes;c_Clostridia;o_Clostridiales;f_Ruminococcaceae;g_	↓	-0.008	0.948	0.927	1

Table S8. Association between exposure to Maternal OW/OB and 3 measures of infant alpha diversity during the first two years of life, both unadjusted and adjusted for exclusive breastfeeding, mode of delivery at birth, antibiotic exposure, and gestational age at birth. Results are estimates from longitudinal hierarchical linear regression models.

		Unadjusted			Adjusted		
**Exposure**	**Infant alpha diversity**	**Estimate**	**95% CI**	**Pr > |t|**	**Estimate**	**95% CI**	**Pr > |t|**
Maternal OW/OB	Observed Species	2.05	(-4.69, 8.78)	0.55	1.89	(-4.79, 8.58)	0.58
	PD Whole Tree	0.15	(-0.31, 0.61)	0.52	0.15	(-0.29, 0.59)	0.49
	Shannon	0.07	(-0.07, 0.22)	0.31	0.07	(-0.07, 0.2)	0.31
Maternal Excessive GWG	Observed Species	1.05	(-6.51, 8.6)	0.79	1.19	(-6.18, 8.57)	0.75
	PD Whole Tree	0.07	(-0.45, 0.6)	0.79	0.13	(-0.37, 0.64)	0.6
	Shannon	-0.01	(-0.17, 0.15)	0.88	-0.01	(-0.17, 0.15)	0.92

Table S9. Results of random forest selection process to identify infant gut microbiota taxa associated with exposure to maternal OW/OB, sorted from most to least important according to the Mean Decrease in Accuracy. The out of bag error was 32.9%. For a similar random forest classifying randomly assigned exposure status, the out of bag error was only 21.6% better, indicating that the model failed to identify significant taxonomic differences with exposure.

OTU_ID	TAXONOMY	0	1	Mean Decrease Accuracy	Mean Decrease Gini
3723096	k_Bacteria; p_Firmicutes; c_Bacilli; o_Lactobacillales; f_Streptococcaceae; g_Streptococcus; s_	0.0112	0.0076	0.0106	22.7806
284014	k_Bacteria; p_Proteobacteria; c_Gammaproteobacteria; o_Enterobacteriales; f_Enterobacteriaceae; g_; s_	0.0074	0.0040	0.0067	23.0879
4425214	k_Bacteria; p_Firmicutes; c_Bacilli; o_Lactobacillales; f_Streptococcaceae; g_Streptococcus; s_	0.0093	-0.0042	0.0067	26.3048
289709	k_Bacteria; p_Proteobacteria; c_Gammaproteobacteria; o_Enterobacteriales; f_Enterobacteriaceae; g_; s_	0.0078	0.0017	0.0067	17.2728
465079	k_Bacteria; p_Firmicutes; c_Bacilli; o_Bacillales; f_Planococcaceae; g_; s_	0.0070	-0.0016	0.0054	15.6910
4481613	k_Bacteria; p_Actinobacteria; c_Coriobacteriia; o_Coriobacteriales; f_Coriobacteriaceae; g_Collinsella; s_aerofaciens	0.0042	0.0079	0.0049	14.1358
511908	k_Bacteria; p_Proteobacteria; c_Gammaproteobacteria; o_Enterobacteriales; f_Enterobacteriaceae; g_Klebsiella; s_	0.0029	0.0092	0.0042	15.6557
997439	k_Bacteria; p_Actinobacteria; c_Actinobacteria; o_Bifidobacteriales; f_Bifidobacteriaceae; g_Bifidobacterium; s_	0.0039	0.0012	0.0034	11.3969
365385	k_Bacteria; p_Actinobacteria; c_Actinobacteria; o_Bifidobacteriales; f_Bifidobacteriaceae; g_Bifidobacterium; s_bifidum	0.0038	0.0006	0.0032	14.7498
180606	k_Bacteria; p_Bacteroidetes; c_Bacteroidia; o_Bacteroidales; f_Bacteroidaceae; g_Bacteroides; s_ovatus	0.0033	0.0001	0.0027	9.9695
3166216	k_Bacteria; p_Firmicutes; c_Bacilli; o_Lactobacillales; f_Streptococcaceae; g_Streptococcus; s_	0.0011	0.0082	0.0025	12.9938
1733364	k_Bacteria; p_Bacteroidetes; c_Bacteroidia; o_Bacteroidales; f_Bacteroidaceae; g_Bacteroides; s_uniformis	0.0013	0.0030	0.0016	9.9213
157748	k_Bacteria; p_Bacteroidetes; c_Bacteroidia; o_Bacteroidales; f_Bacteroidaceae; g_Bacteroides; s_	0.0013	0.0013	0.0013	5.2361
2325032	k_Bacteria; p_Firmicutes; c_Clostridia; o_Clostridiales; f_Clostridiaceae; g_; s_	0.0002	0.0043	0.0010	12.5667
2599028	k_Bacteria; p_Bacteroidetes; c_Bacteroidia; o_Bacteroidales; f_Bacteroidaceae; g_Bacteroides; s_	-0.0004	0.0054	0.0007	5.7813
328472	k_Bacteria; p_Actinobacteria; c_Actinobacteria; o_Actinomycetales; f_Actinomycetaceae; g_Varibaculum; s_	0.0000	0.0026	0.0005	6.6155
3449122	k_Bacteria; p_Firmicutes; c_Bacilli; o_Lactobacillales; f_Streptococcaceae; g_Streptococcus; s_	-0.0023	0.0084	-0.0002	10.3829
4294457	k_Bacteria; p_Actinobacteria; c_Actinobacteria; o_Actinomycetales; f_Micrococcaceae; g_Rothia; s_mucilaginosa	-0.0024	0.0070	-0.0006	13.9014

Table S10. Results of random forest selection process to identify infant gut microbiota taxa associated with exposure to excessive GWG, sorted from most to least important according to the Mean Decrease in Accuracy. The out of bag error was 36%. For a similar random forest classifying randomly assigned exposure status, the out of bag error was only 8% better, indicating that the model failed to identify significant taxonomic differences with exposure.

OTU_ID	TAXONOMY	0	1	Mean Decrease Accuracy	Mean Decrease Gini
681370	k_Bacteria; p_Actinobacteria; c_Actinobacteria; o_Bifidobacteriales; f_Bifidobacteriaceae; g_Bifidobacterium; s_pseudolongum	0.0166	0.0069	0.0115	17.4474
4449054	k_Bacteria; p_Bacteroidetes; c_Bacteroidia; o_Bacteroidales; f_Bacteroidaceae; g_Bacteroides; s_	0.0097	0.0030	0.0062	11.5553
284014	k_Bacteria; p_Proteobacteria; c_Gammaproteobacteria; o_Enterobacteriales; f_Enterobacteriaceae; g_; s_	0.0071	0.0050	0.0060	22.1594
510870	k_Bacteria; p_Proteobacteria; c_Gammaproteobacteria; o_Enterobacteriales; f_Enterobacteriaceae; g_; s_	0.0066	0.0040	0.0052	15.5170
4458700	k_Bacteria; p_Firmicutes; c_Clostridia; o_Clostridiales; f_Lachnospiraceae; g_[Ruminococcus]; s_	0.0083	0.0015	0.0047	8.3807
3903651	k_Bacteria; p_Firmicutes; c_Clostridia; o_Clostridiales; f_Ruminococcaceae; g_Oscillospira; s_	0.0099	-0.0013	0.0040	8.8141
272150	k_Bacteria; p_Firmicutes; c_Bacilli; o_Lactobacillales; f_Streptococcaceae; g_Streptococcus; s_	0.0001	0.0071	0.0037	21.6168
4347159	k_Bacteria; p_Actinobacteria; c_Actinobacteria; o_Bifidobacteriales; f_Bifidobacteriaceae; g_Bifidobacterium; s_adolescentis	0.0028	0.0043	0.0035	21.9004
540402	k_Bacteria; p_Proteobacteria; c_Gammaproteobacteria; o_Enterobacteriales; f_Enterobacteriaceae; g_; s_	0.0032	0.0031	0.0031	8.4222
511908	k_Bacteria; p_Proteobacteria; c_Gammaproteobacteria; o_Enterobacteriales; f_Enterobacteriaceae; g_Klebsiella; s_	0.0052	0.0012	0.0031	8.6857
109382	k_Bacteria; p_Proteobacteria; c_Betaproteobacteria; o_Burkholderiales; f_; g_; s_	0.0014	0.0044	0.0030	12.7347
198449	k_Bacteria; p_Bacteroidetes; c_Bacteroidia; o_Bacteroidales; f_Bacteroidaceae; g_Bacteroides; s_	0.0026	0.0031	0.0029	10.1082
1061772	k_Bacteria; p_Firmicutes; c_Bacilli; o_Bacillales; f_Planococcaceae; g_; s_	0.0003	0.0050	0.0027	12.0601
2599028	k_Bacteria; p_Bacteroidetes; c_Bacteroidia; o_Bacteroidales; f_Bacteroidaceae; g_Bacteroides; s_	0.0013	0.0040	0.0027	6.0859
183089	k_Bacteria; p_Firmicutes; c_Clostridia; o_Clostridiales; f_Clostridiaceae; g_; s_	-0.0002	0.0050	0.0025	10.3876
941632	k_Bacteria; p_Actinobacteria; c_Actinobacteria; o_Actinomycetales; f_Corynebacteriaceae; g_Corynebacterium; s_	0.0020	0.0028	0.0024	8.6352
1142029	k_Bacteria; p_Actinobacteria; c_Actinobacteria; o_Bifidobacteriales; f_Bifidobacteriaceae; g_Bifidobacterium; s_	0.0022	0.0026	0.0024	18.7387
932077	k_Bacteria; p_Actinobacteria; c_Actinobacteria; o_Actinomycetales; f_Micrococcaceae; g_Rothia; s_mucilaginosa	0.0021	0.0022	0.0022	8.0013
4376828	k_Bacteria; p_Actinobacteria; c_Actinobacteria; o_Bifidobacteriales; f_Bifidobacteriaceae; g_Bifidobacterium; s_	0.0041	-0.0002	0.0018	8.9619
4434334	k_Bacteria; p_Firmicutes; c_Clostridia; o_Clostridiales; f_Clostridiaceae; g_; s_	0.0013	0.0011	0.0012	11.3705
518002	k_Bacteria; p_Proteobacteria; c_Gammaproteobacteria; o_Enterobacteriales; f_Enterobacteriaceae; g_; s_	0.0015	0.0005	0.0010	3.8294
4425214	k_Bacteria; p_Firmicutes; c_Bacilli; o_Lactobacillales; f_Streptococcaceae; g_Streptococcus; s_	0.0016	-0.0011	0.0002	21.3768

Supplemental Figures

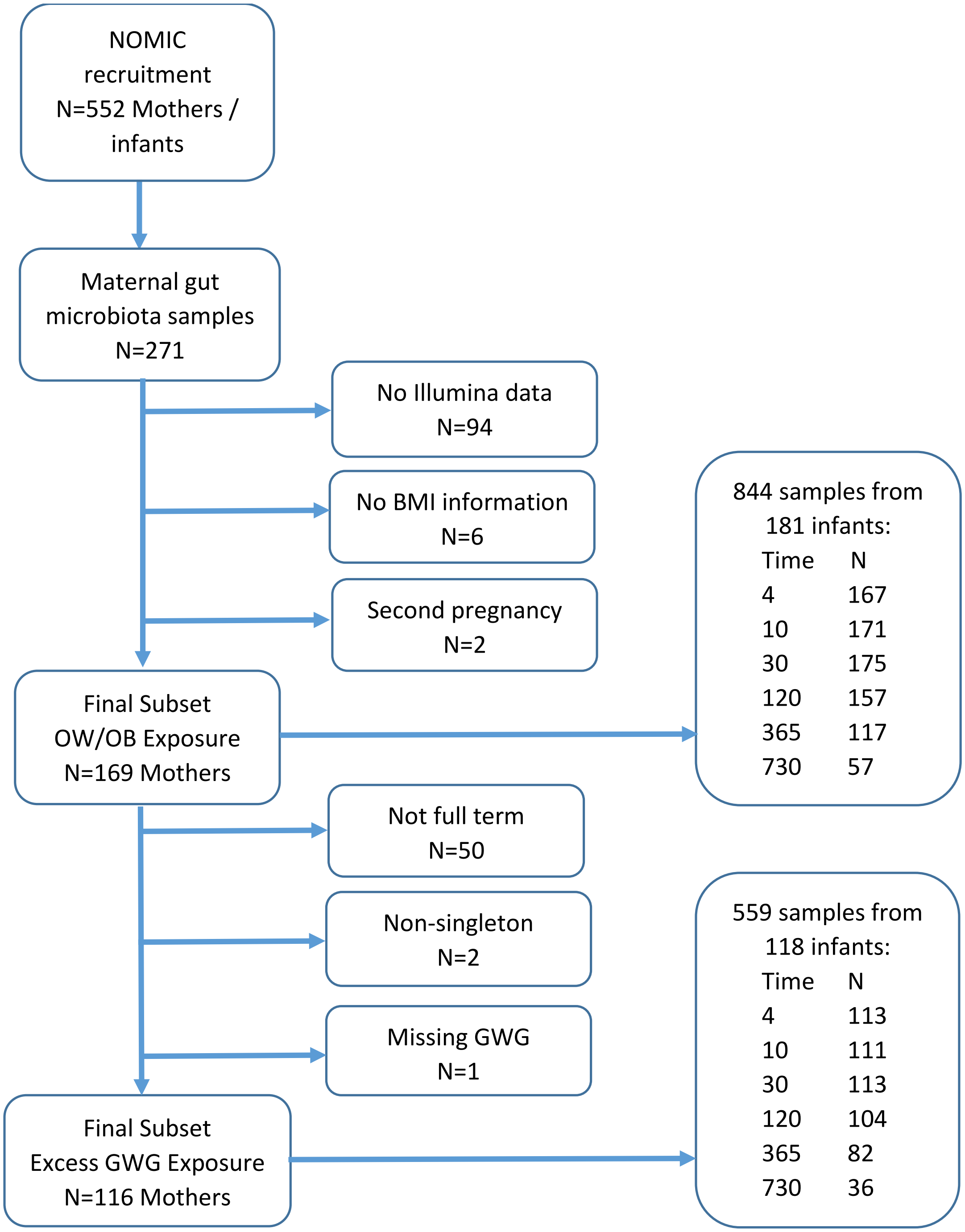

Figure S3. Flow chart for the samples from the NoMIC cohort included in this study.

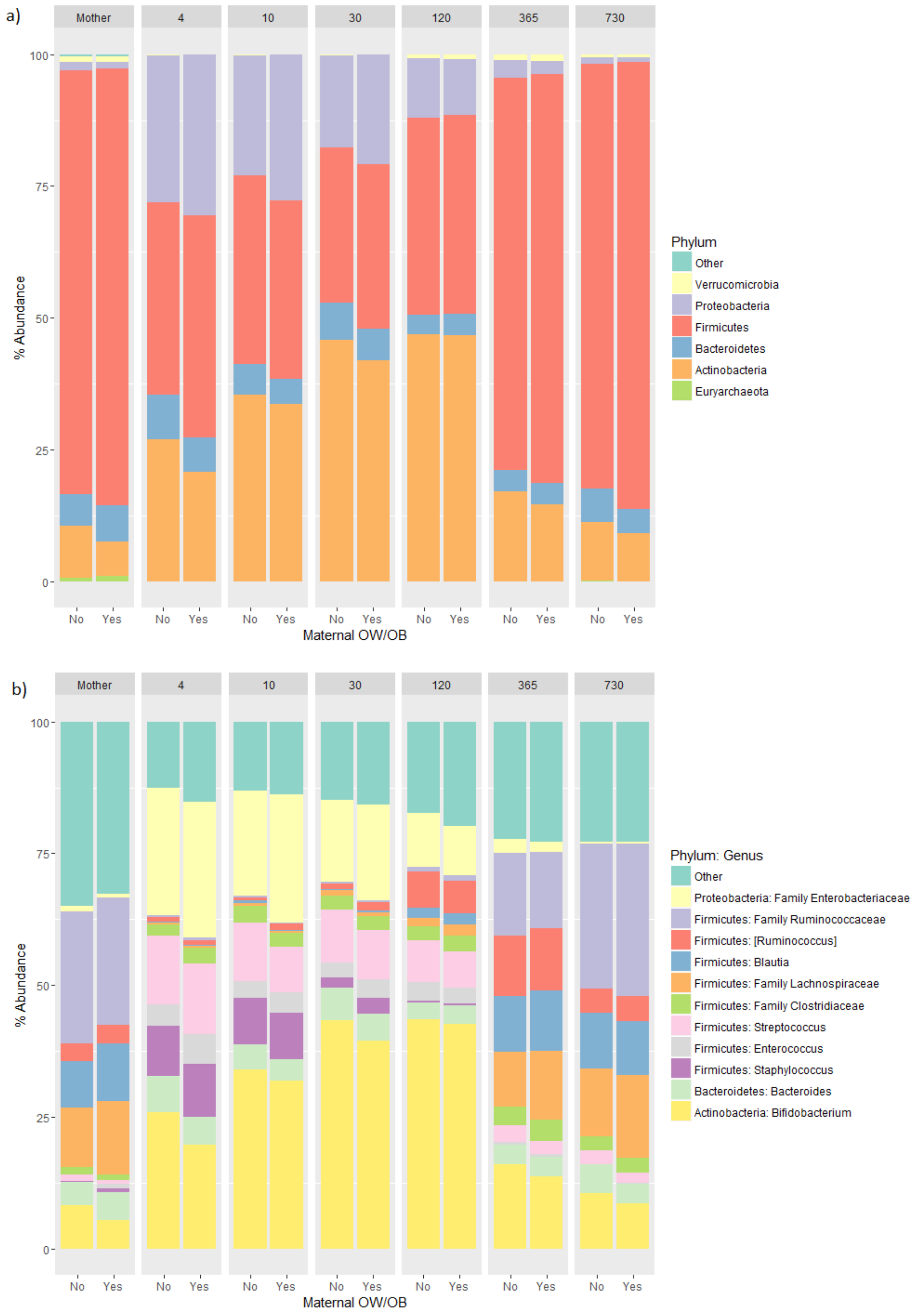

Figure S4. Most abundant a) phylum and b) genus level taxa in maternal samples at the time of delivery and infant samples at days 4, 10, 30, 120, 365, and 730 post-birth by maternal pre-pregnancy overweight / obese (OW/OB) status.

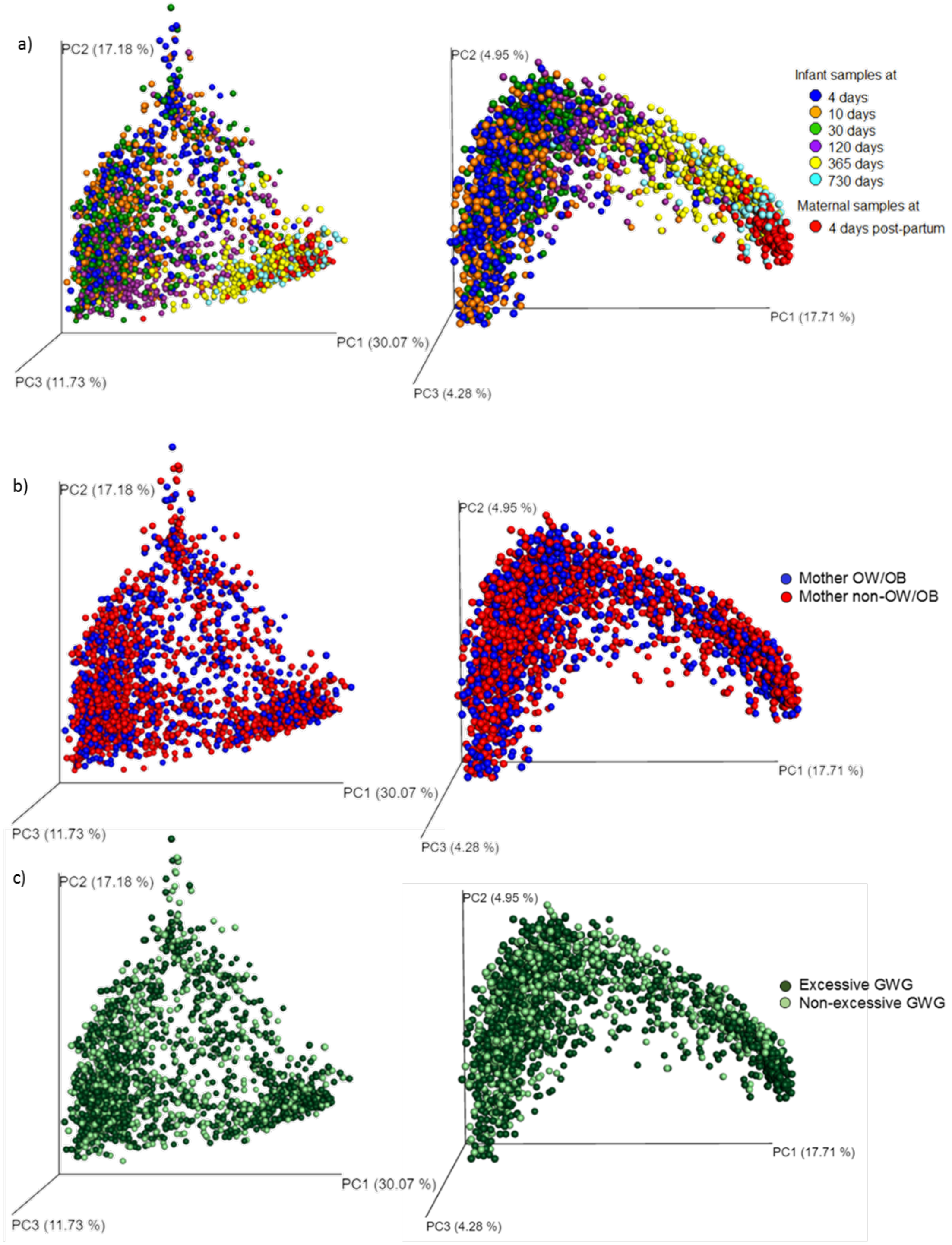

Figure S5. Principal coordinate analysis plots of weighted (left) and unweighted (right) UniFrac distance of maternal gut microbiota samples 4 days post-partum and infant gut samples at days 4, 10, 30, 120, 365, and 730 post-birth by a) sampling time, b) maternal overweight / obesity (OW/OB) and c) maternal excessive gestational weight gain (GWG).
